# Angiotensin 1-7 Protects against Angiotensin II-Induced Endoplasmic Reticulum Stress and Endothelial Dysfunction via Mas Receptor

**DOI:** 10.1371/journal.pone.0145413

**Published:** 2015-12-28

**Authors:** Dharmani Murugan, Yeh Siang Lau, Wai Chi Lau, Mohd Rais Mustafa, Yu Huang

**Affiliations:** 1 Department of Pharmacology, Faculty of Medicine, University of Malaya, Kuala Lumpur 50603, Malaysia; 2 Li Ka Shing Institute of Health Sciences, Chinese University of Hong Kong, Hong Kong, China; Nagoya University, JAPAN

## Abstract

Angiotensin 1–7 (Ang 1–7) counter-regulates the cardiovascular actions of angiotensin II (Ang II). The present study investigated the protective effect of Ang 1–7 against Ang II-induced endoplasmic reticulum (ER) stress and endothelial dysfunction. *Ex vivo* treatment with Ang II (0.5 μM, 24 hours) impaired endothelium-dependent relaxation in mouse aortas; this harmful effect of Ang II was reversed by co-treatment with ER stress inhibitors, l4-phenylbutyric acid (PBA) and tauroursodeoxycholic acid (TUDCA) as well as Ang 1–7. The Mas receptor antagonist, A779, antagonized the effect of Ang 1–7. The elevated mRNA expression of CHOP, Grp78 and ATF4 or protein expression of p-eIF2α and ATF6 (ER stress markers) in Ang II-treated human umbilical vein endothelial cells (HUVECs) and mouse aortas were blunted by co-treatment with Ang 1–7 and the latter effect was reversed by A779. Furthermore, Ang II-induced reduction in both eNOS phosphorylation and NO production was inhibited by Ang 1–7. In addition, Ang 1–7 decreased the levels of ER stress markers and augmented NO production in HUVECs treated with ER stress inducer, tunicamycin. The present study provides new evidence for functional antagonism between the two arms of the renin-angiotensin system in endothelial cells by demonstrating that Ang 1–7 ameliorates Ang II-stimulated ER stress to raise NO bioavailability, and subsequently preserves endothelial function.

## Introduction

The endoplasmic reticulum (ER) is the key cell organelle responsible for protein translation, folding and trafficking. The maintenance of calcium homeostasis, and production and storage of glycogen as well as other macromolecules take place in the ER which is also the early site responding to cellular stress [1, 2, 3]. Disruption in ER homeostasis or function is associated with oxidative stress, inflammatory reaction, hyperglycemia, calcium deprivation, and the exposure to chemicals such as thapsigargin or tunicamycin leads to misfolding and aggregation of proteins within ER lumen; a process known as ER stress, leading to activation of a complex signaling network called the unfolded protein response (UPR), which attempts to restore normal ER function [1, 4]. The UPR is activated by 3 main signaling pathways: (1) the inositol-requiring protein 1 (IRE-1) activation, (2) the protein kinase RNA–like ER kinase (PERK) activation, and (3) the activating transcription factor 6 (ATF6) [1]. Recent evidence indicates the involvement of ER stress in diabetes, hypertension, cardiac hypertrophy, atherosclerosis, and ischemic heart disease [5, 6, 7, 8].

The renin-angiotensin system (RAS) is an important regulator of cardiovascular homeostasis and its major peptide, angiotensin II (Ang II) promotes vasoconstriction, inflammation, salt and water reabsorption, and oxidative stress. The actions and expression of angiotensin receptors are elevated in cardiovascular related diseases [9, 10]. Angiotensin receptor type 1 (AT1R) blockers inhibit ER stress in the heart [11, 12, 13] and kidney of streptozotocin-induced diabetic rats [14], suggesting a link between the harmful arm of the RAS and ER stress induction. This notion is further supported by recent observations that inhibitors of ER stress are able to inhibit Ang II-induced hypertension [2, 8].

Angiotensin 1–7 (Ang 1–7), a heptapeptide of the RAS has been demonstrated to counter-regulate the actions of Ang II and to protect cardiovascular function through enhancing vasodilatation via elevated release of NO and bradykinin, as well as inhibiting production of reactive oxygen species (ROS) [15, 16, 17]. Although Ang 1–7 is reported to be protective to the cardiovascular function, it is still largely unclear whether this involves inhibition of ER stress. Thus, the present study aims to investigate the beneficial effect of Ang 1–7 against Ang II-induced ER stress and endothelial dysfunction.

## Methods

### 2.1 Animals and experimental protocol

Male C57BL/6J mice (10–11 weeks old) were purchased from the Laboratory Animal Service Center of Chinese University of Hong Kong (CUHK). The experimental procedures were approved by The Chinese University of Hong Kong (CUHK) Animal Experimentation Ethics Committee, conforming to National Institute of Health (NIH) guidelines. Mice were maintained in a well-ventilated holding room at constant temperature of 24 ± 1°C and received normal chow and tap water *ad libitum*.

### 2.2 Organ culture of aortas and force measurements

Mice were sacrificed by CO_2_ inhalation and the thoracic aortas were isolated, cleaned of surrounding connective tissues, and cut into rings segments of ~2 mm in length. Rings were cultured in serum-free Dulbeco’s Modified Eagle’s Media (DMEM, Gibco, Gaithersberg, MD) supplemented with 10% fetal bovine serum (FBS, Gibco), 100 U/ml penicillin and 100 μg/ml streptomycin. Rings were exposed to Ang II (0.5 μM) for 24 hours in 5% CO_2_ incubator at 37°C and co-treated with ER stress inhibitors (4-phenylbutyric acid (PBA, 10 μM) or tauroursodeoxycholic acid (TUDCA, 20 μM)); AT1R antagonist losartan (3 μM); superoxide dismutase mimetic tempol (100 μM); Ang 1–7 (0.1–10 μM); Mas receptor antagonist A779 (10 μM) plus Ang 1–7 (1 μM). All the co-incubated drugs were added 30 minutes before the addition of Ang II while A779 was added 10 minutes before Ang 1–7. At the end of incubation period, rings were suspended, maintained at 37°C, and stretched to an optimal tension of 3 mN in a Myograph System (Danish Myo Technology, Aarhus, Denmark), and continuously oxygenated with 95% O_2_ and 5% CO_2_. The changes of isometric tension were recorded using the PowerLab LabChart 6.0 recording system (AD Instruments, New South Wales, Australia) [18,19]. Each experiment was conducted with separate rings from a minimum of 5–6 mice. Some arteries were snap-frozen in liquid nitrogen following organ culture and stored in -80°C for later processing.

### 2.3 Experimental protocol

After 60 minute equilibration, rings were first contracted with 60 mM KCl and washed three times in Krebs solution before phenylephrine (1 μM) was added to induce a sustained contraction. Concentration-response curves for endothelium-dependent relaxations to acetylcholine (ACh, 3 nM to 10 μM) or for endothelium-independent relaxations to sodium nitroprusside (SNP, 1 nM to 10 μM) were constructed.

### 2.4 Cell culture

Human umbilical vein endothelial cells (HUVECs, American Type Culture Collection (ATTC), Manassas, USA) were cultured in EGM-2 medium (EGM-2, Single Quots, Lonza) containing 20% fetal bovine serum. HUVECs were grown in 12- or 6-well chambers. After 80–90% confluence was obtained, they were treated with Ang II (0.5 μM) for 24 hours and co-incubated with PBA (10 μM), TUDCA (20 μM), losartan (3 μM), tempol (100 μM), Ang 1–7 (1 μM) or A779 (10 μM) plus Ang 1–7. To determine the effect of Ang 1–7 on ER stress inducer, tunicamycin, another set of experiments was repeated in HUVECs exposed to tunicamycin (2 μg/mL) for 16 hours with or without Ang 1–7 (1 μM) plus A779 (10 μM).

### 2.5 Western blotting

HUVECs and mouse aortas were homogenized in ice-cold 1X RIPA buffer containing leupeptin 1 μg/ml, aprotonin 5 μg/ml, PMSF 100 μg/ml, sodium orthovanadate 1 mM, EGTA 1 mM, EDTA 1 mM, NaF 1 mM, and β-glycerolphosphate 2 mg/ml. The lysates were centrifuged at 20 000 x g for 20 minutes and the supernatant was collected for Western blotting. Protein concentrations were determined by modified Lowry assay (Bio-Rad Laboratories, Hercules, CA, USA). Total protein concentration of 15 μg for each lane was separated in 10% sodium dodecyl sulphate (SDS)-polyacrylamide gel and then transferred to an immobilon-P polyvinylidene difluoride (PVDF) membrane (Millipore, Billerica, MA, USA) at 100 V. The blots were blocked for non-specific binding by 3% bovine serum albumin (BSA) or 5% non-fat milk in Tris-buffered saline containing 0.1% Tween 20 (TBS) with gentle shaking. Following blocking, the blots were incubated with either primary polyclonal anti-eIF2α (1:500, Cell Signaling), anti-peIF2α (1:500, Invitrogen), anti-ATF6 (1:500, Abcam), anti- eNOS at Ser1177 (p-eNOSSer1177) (1:1000, Abcam), monoclonal anti-eNOS (1:1000, BD Transduction Laboratory). After overnight incubation at 4°C, the membranes were washed and incubated with respective secondary antibodies conjugated to horseradish peroxidase for 2 hours at room temperature. The membranes were then developed with enhanced chemiluminescence detection system (ECL reagents, Cell Signalling) and exposed on X-ray films. Protein expression was quantified by densitometer (FluorChem; Alpha Innotech, San Leandro, CA, USA), normalized to GAPDH for ATF6, eNOS for peNOS, eIF2α for p- eIF2α and then compared with control.

### 2.6 Real-time PCR

Total RNA was extracted from HUVECs using the TRIzol reagent (Ambion, Life Technologies). A 100 ng of total RNA was used for cDNA synthesis using cDNA reverse transcription kit (ABI Research). The real-time PCR, which was contained in a final volume of 11 μL, consisted of 1 μL cDNA and components of SYBR-Green (Applied Biosystems). The PCR was carried out on 384-well plates using the ABI Viia TM 7 Detection System. The following primers were used to detect changes in mRNA levels of ER stress genes: CHOP (116bp), the primer sequences were 5’- GGAAACAGAGTGGTCATTCCC-3’ (forward) and 5’-CTGCTTGAGCCGTTCATTCTC-3’(reverse); for Grp78 (167bp), 5’TCTGCTTGATGTGTGTCCTCTT (forward) and 5’- GTCGTTCACCTTCGTAGACCT-3’ (reverse); for ATF4 (165bp), 5’- CTCCGGGACAGATTGGATGTT-3’ (forward) and 5’- GGCTGCTTATTAGTCTCCTGGAC-3’ (reverse); for GAPDH, the primer sequences were 5’- GGAGCGAGATCCCTCCAAAAT-3’ (forward) and 5’- GGCTGTTGTCATACTTCTCATGG-3’. Relative expression levels were calculated from 2ΔC^t^, normalized to GAPDH and then compared with control.

### 2.7 Detection of NO production in HUVECs

The previous study described the method used to assay NO levels [20]. Briefly, the confluent HUVECs were seeded on the coverslip and followed by treatment with Ang II with or without Ang 1–7 and A779 for 24 hours. Another set of experiments was repeated following exposure to tunicamycin with or without Ang 1–7 and A779 for 16 hours. At the end of the treatment period, HUVECs were rinsed in NPSS and incubated in 1 μM 4-amino-5-methylamino-2’,7’-difluorofluorescein (DAF-FM diacetate, Molecular Probes) for 10 minutes at 37°C. NO production stimulated by calcium ionophore A23187 (1 μM) was measured as reflected by changes in fluorescence intensity under the Olympus Fluoview FV1000 laser scanning confocal system (Olympus America, Inc., Melville, NY, USA) mounted on an inverted IX81 Olympus microscope with excitation at 495 nM and emission at 515 nM. The results were presented as a ratio of fluorescence intensity (F1/F0) before and after the addition of A23187.

### 2.8 Statistical analysis

Results are means ± standard error of the mean (S.E.M). The n denotes the number of aortic rings isolated from different mice in functional studies or the number of wells used in culture studies. Concentration-response curves were fitted to a sigmoidal curve using non-linear regression with the aid of the statistical software GraphPad Prism version 6, (USA). Data were analyzed for statistical significance using Student’s t-test for unpaired observations and one and two-way analysis of variance (ANOVA) followed by Newman-Keul’s multiple comparison test for multiple group comparisons (Prism 6.0, GraphPad Software, USA). p<0.05 was taken as statistically significant.

## Results

### 3.1 ER stress inhibitors reversed Ang II-impaired endothelium-dependent relaxations

**Fig 1:**
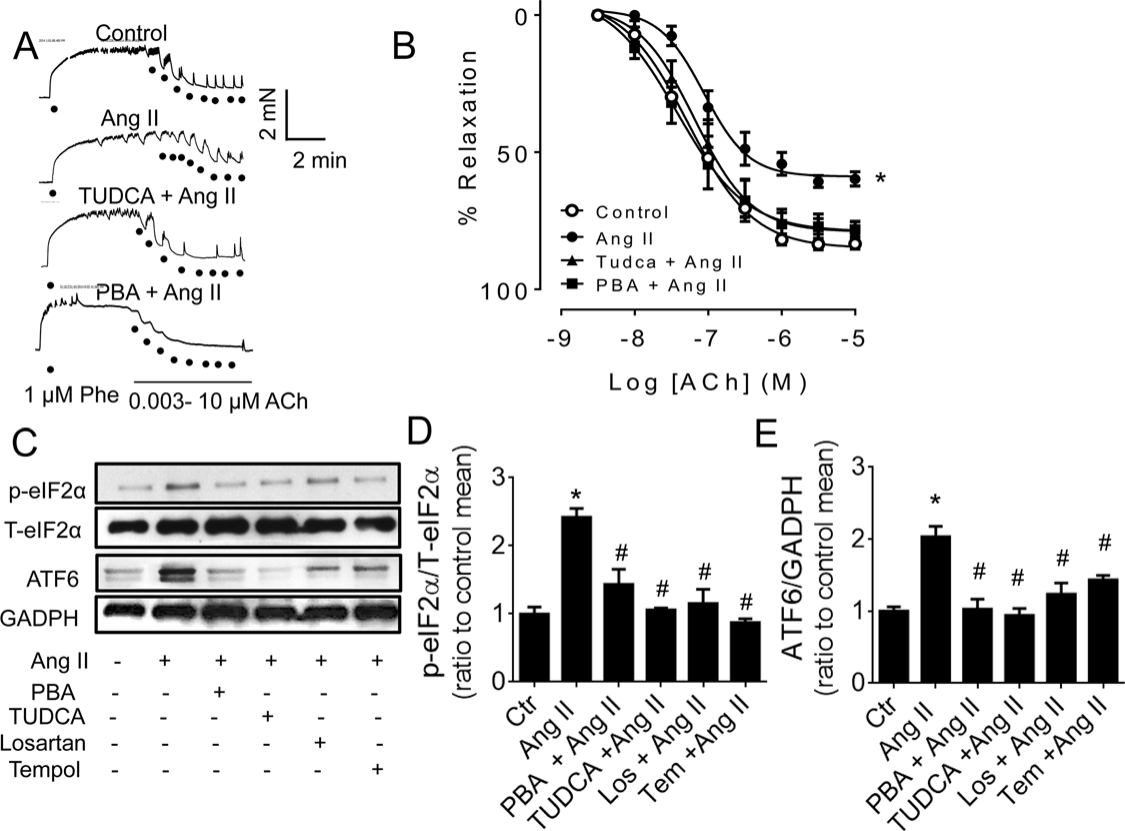
Ang II-induced impairment of ACh-induced endothelium-dependent relaxations (EDR) was reversed by co-treatment with ER stress inhibitors, PBA and TUDCA in mouse aortas. Representative traces (A) and summarized graph (B) showing Ang II (0.5 μM, 24 hours) attenuated EDR but reversed by a combined exposure to PBA (10 μM) and TUDCA (20 μM). Data are means ± S.E.M (n = 5–6) (C) Representative blots and relative expression of ER stress proteins, (D) phosphorylated eIF2α and (E) ATF6 in HUVECs exposed to Ang II with and without PBA (10 μM), TUDCA (20 μM), tempol (100 μM) or losartan (3 μM). Data are mean ± S.E.M (n = 5). ^*^p<0.05 vs control; #p<0.05 vs Ang II.

*Ex vivo* 24-h treatment of mouse aortas with Ang II (0.5 μM) attenuated endothelium-dependent relaxations (EDR, Fig 1A and 1B) and this EDR impairment was reversed by co-treatment with ER stress inhibitors, PBA and TUDCA (Fig 1A and 1B and Table 1). In contrast, SNP-induced endothelium-independent relaxations were comparable in all groups (Figure A in S1 File). Serving as positive controls, co-treatment with either losartan or tempol reversed the effect of Ang II on EDR (Figure B in S1 File). TUDCA, PBA and Losartan alone did not affect the relaxation to ACh (Figure C in S1 File).

**Table 1:** The agonist sensitivity (pEC_50_) and maximum effect (E_max_) of ACh-induced relaxations in mouse aortas following pre-treatment with Ang II with and without PBA (10 μM), TUDCA (20 μM), tempol (100 μM) or losartan (3 μM). Data are mean ± S.E.M (n = 5–6).

	ACh-induced relaxations	
Group	pEC_50_ (-log M)	E_max_ (%)
Control	7.27 ± 0.07	85.01 ± 2.08
Ang II	7.06 ± 0.08	59.78 ± 2.62 ^*^
TUDCA + Ang II	7.19 ± 0.11	78.87 ± 3.61 ^#^
PBA + Ang II	7.42 ± 0.18	78.14 ± 2.78 ^#^
Losartan + Ang II	7.26 ± 0.07	79.53 ± 2.86 ^#^
Tempol + Ang II	7.15 ± 0.05	77.84 ± 1.48 ^#^

^*^p<0.05 vs control

#p<0.05 vs Ang II.

### 3.2 ER stress inhibitors reversed Ang II-induced ER stress in HUVECs

The levels of ER stress proteins, p-eIF2α and ATF6 were significantly elevated in Ang II-treated HUVECs, which was normalized by co-treatment with PBA, TUDCA, losartan, and tempol (Fig 1C, 1D and 1E).

### 3.3 Ang 1–7 reversed Ang II-induced endothelial dysfunction

**Fig 2:**
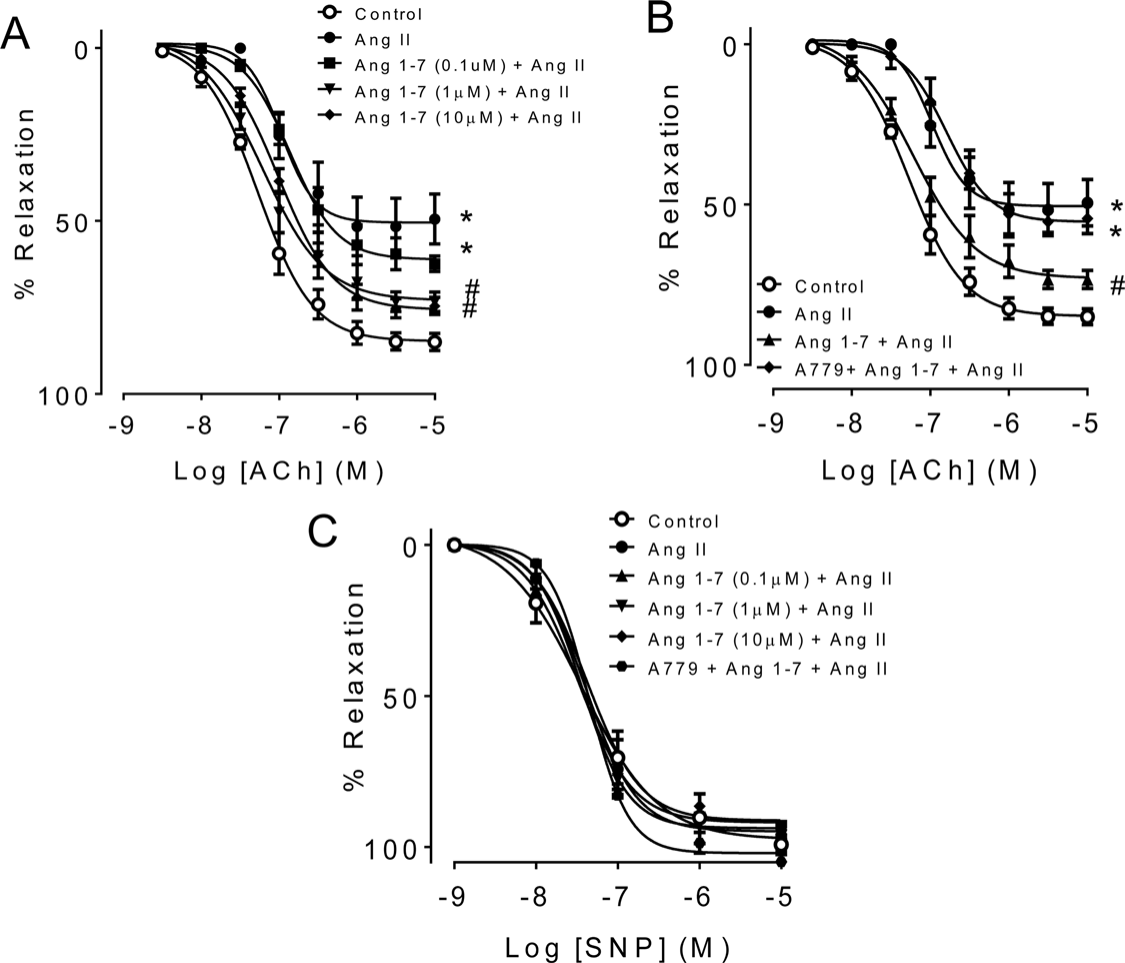
Ang 1–7 improved EDR in Ang II-treated mouse aortas. (A) Concentration- response curve to ACh-induced EDR following 24-hour treatment with Ang II (0.5 μM) in the presence of Ang 1–7 (0.1–10 μM). (B) Co-incubation with the Mas receptor antagonist, A779 (10 μM) reversed the effect of Ang 1–7. (C) SNP-induced relaxations were similar in all groups. Data are mean ± S.E.M (n = 5–6). ^*^p<0.05 vs control; #p<0.05 vs Ang II.

Treatment with Ang 1–7 (1 and 10 μM) rescued the Ang II-impaired EDR (Fig 2A and Table 2) while co-incubation with A779, Mas receptor antagonist reversed the effect of Ang 1–7 (Fig 2B and Table 2). By contrast, SNP-induced relaxations were similar in all groups (Fig 2C).

**Table 2:** The agonist sensitivity (pEC_50_) and maximum effect (E_max_) of ACh-induced relaxations in mouse aortas following pre-treatment with Ang II with and without presence of various concentrations of Ang 1–7 (0.1–10 μM). Data are mean ± S.E.M (n = 5–6).

	ACh-induced relaxations	
Group	pEC_50_(-log M)	E_max_ (%)
Control	7.27 ± 0.06	84.99 ± 2.48
Ang II	6.98 ± 0.12	49.44 ± 7.23 ^*^
Ang 1–7 (0.1 μM) + Ang II	6.96 ± 0.08	62.41 ± 2.24^*^
Ang 1–7 (1 μM) + Ang II	7.21 ± 0.10	73.40 ± 2.83 ^#^
Ang 1–7 (10 μM) + Ang II	7.01 ± 0.05	74.58 ± 2.50 ^#^
A779 + Ang 1–7 (1 μM) + Ang II	6.89 ± 0.19	54.29 ± 4.77 ^*^

^*^p<0.05 vs control

#p<0.05 vs Ang II.

### 3.4 Ang 1–7 decreased ER stress stimulated by Ang II and tunicamycin

**Fig 3:**
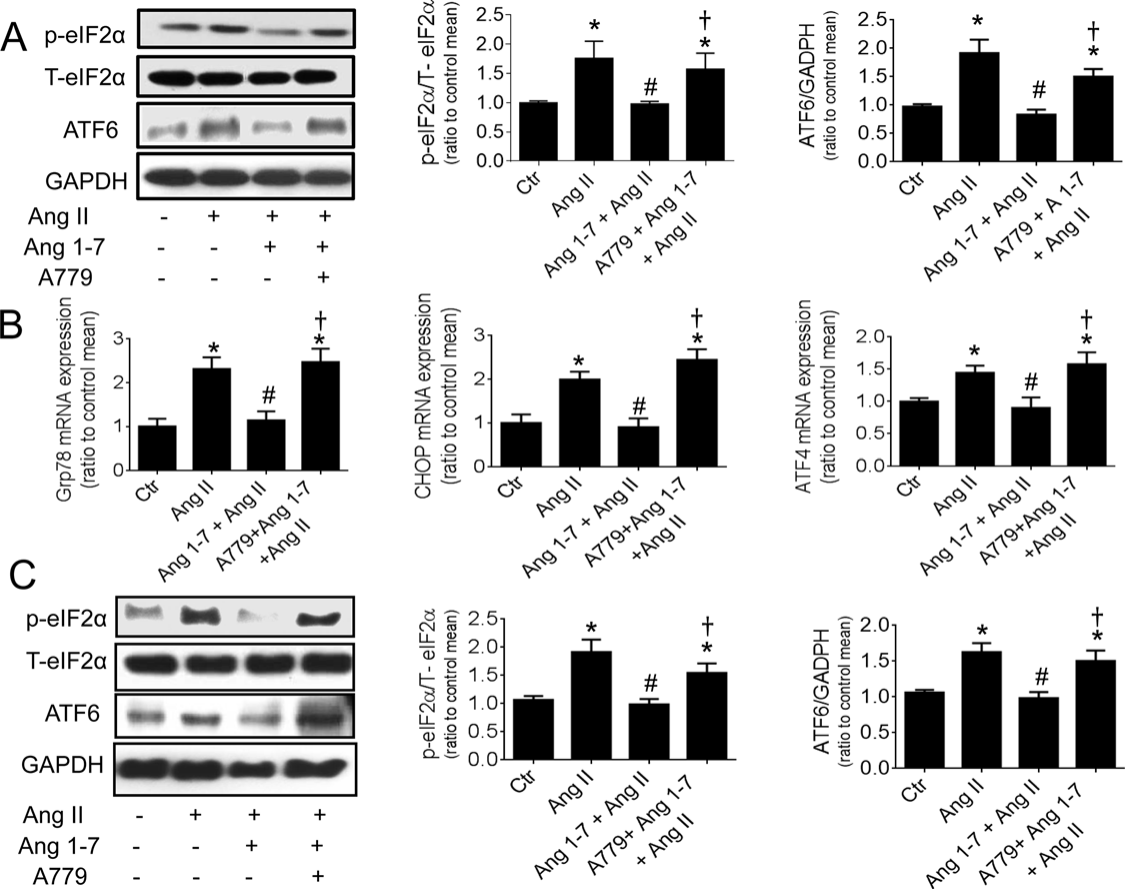
Ang 1–7 decreased Ang II-stimulated ER stress. Representative blots and relative expression of ER stress proteins, phosphorylated eIF2α and ATF6 in (A) HUVECs and (C) in mouse aortas exposed to Ang II (0.5 μM, 24 hours) and co-treated with Ang 1–7 (1μM) and A779 (10 μM). (B) The mRNA expression of ER stress genes, CHOP, Grp78 and ATF4 in Ang II-treated HUVECs with or without Ang 1–7 and A779. Data are mean ± S.E.M (n = 5). ^*^p<0.05 vs control; #p<0.05 vs Ang II, † p<0.05 vs Ang 1–7+Ang II.

**Fig 4:**
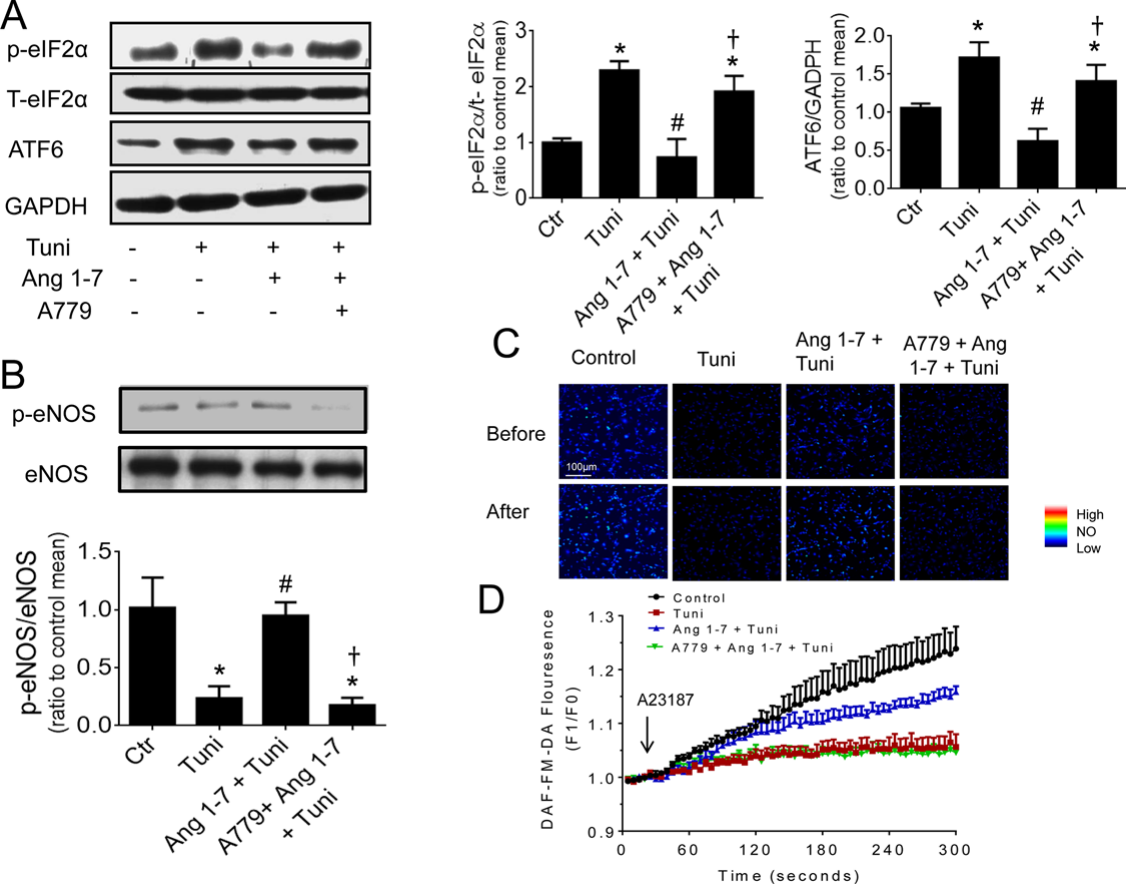
Ang 1–7 decreased tunicamycin-stimulated ER stress and improved NO bioavailability in HUVECS. Representative blots and relative expression of phosphorylated eIF2α and ATF6 (A) and phosphorylated eNOS (B) in HUVECs exposed to tunicamycin (2 μg/mL, 16 hours) and co-treated with Ang 1–7 (1 μM) and A779 (10 μM). Representative images (C) and summarized results (D) for NO production in HUVECs under different treatments. Data are mean ± S.E.M (n = 5–6). ^*^p<0.05 vs control; #p<0.05 vs tunicamycin, † p<0.05 vs Ang 1–7+Ang II.

The expression of ER stress proteins, p-eiF2α and ATF6 were elevated by Ang II in HUVECs. This elevation was reversed by co-incubation with Ang 1–7 (1 μM) while the presence of A779 antagonized the effect of Ang 1–7 (Fig 3A). Ang II-induced increase in the mRNA expression of CHOP and Grp78 (ER chaperones) and ATF4 was also inhibited by Ang 1–7, which was again antagonized by A779 (Fig 3B). Likewise, Ang II-treated mouse aortas exhibited an elevated protein content for p-eIF2α and ATF6, which was reversed by Ang 1–7 (Fig 3C). In addition, Ang 1–7 suppressed tunicamycin-stimulated rise in p-eiF2α and ATF6 and this effect was also reversed by A779 (Fig 4A).

### 3.5 Ang 1–7 increased eNOS phosphorylation and NO production

**Fig 5:**
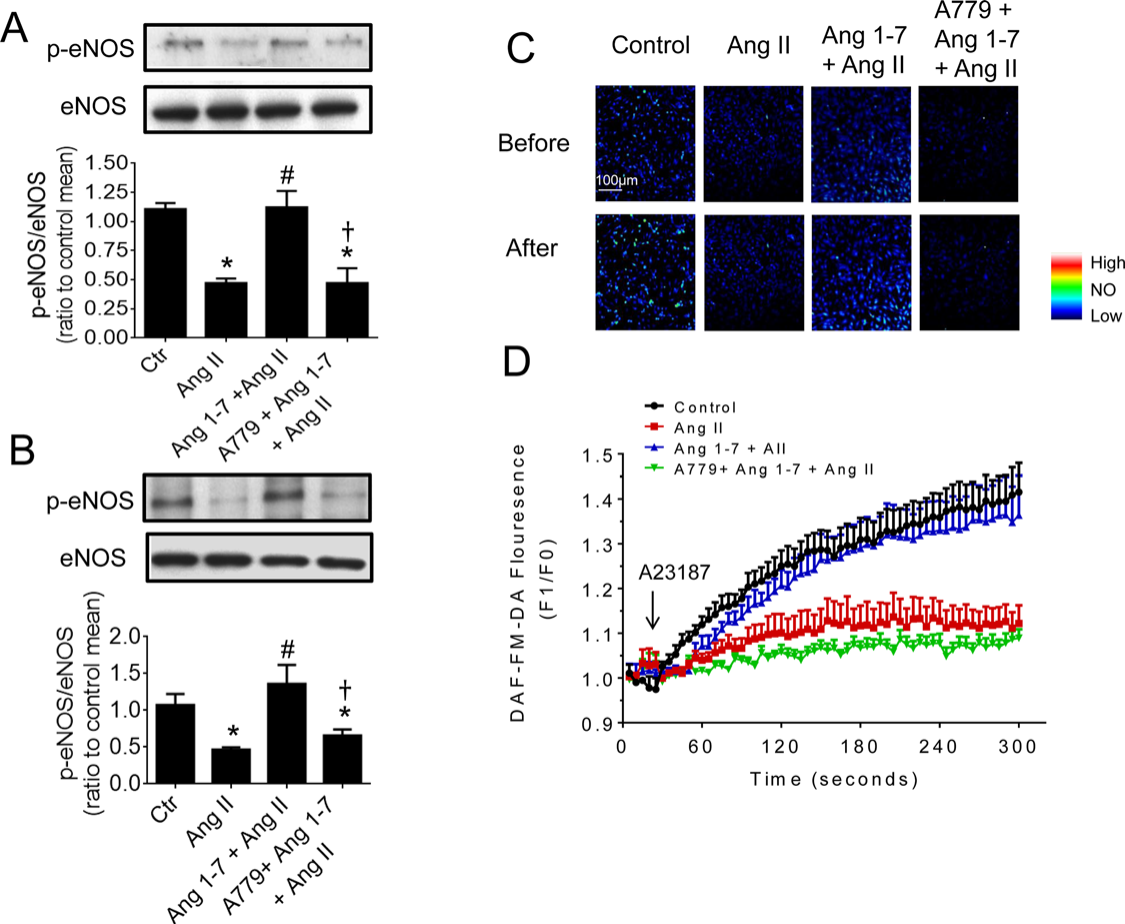
Ang 1–7 increased phosphorylated eNOS at Ser1177 and NO production. Representative blots and relative protein expression of phosphorylated eNOS in (A) HUVECs and (B) in mouse aortas exposed to Ang II (0.5 μM, 24 hours) and co-treated with Ang 1–7 (1 μM) and A779 (10 μM). Data are means ± S.E.M (n = 5–6). Representative images (C) and summarized results (D) of fluorescence imaging of NO production using 4-amino-5-methylamino-2',7'-difluorofluorescein (DAF-FM) in HUVECs under different treatments. Data are mean ± S.E.M (n = 5–6). ^*^p<0.05 vs control; #p<0.05 vs Ang II, † p<0.05 vs Ang 1–7+Ang II.

eNOS phosphorylation was decreased in HUVECs and aortas following Ang II exposure (Fig 5A and 5B) and in tunicamycin-treated HUVECs (Fig 4B). Ang 1–7 co-treatment restored the lost eNOS phosphorylation and this effect was reversed by A779 (Fig 5A and 5B). Furthermore, A23187-stimulated NO production in HUVECs was decreased by Ang II and tunicamycin. Ang 1–7 treatment restored the NO production that was diminished by Ang II (Fig 5C and 5D). Again, A779 reversed the effect of Ang 1–7. Finally, Ang 1–7 preserved NO production in tunicamycin-treated HUVECs (Fig 4C and 4D) and this effect was antagonized by A779.

## Discussion

The present study investigated the role of Ang 1–7 in ameliorating ER stress and protects endothelial function against Ang II insult in mouse arteries and human endothelial cells. The present findings demonstated that (1) Ang II-stimulated ER stress contributes to endothelial dysfunction; (2) Ang 1–7 inhibits Ang II-stimulated ER stress and is most likely mediated via Mas receptor and (3) Ang 1-7-induced inhibition of ER stress augments NO bioavailability and thus restores the impaired EDR.

ER stress is an active contributor to endothelial dysfunction. *Ex vivo* culture of mouse aortas with ER stress inducer, tunicamycin impairs EDR, thus providing direct evidence that ER stress damages endothelial function [20]. The present study demonstrates that *ex vivo* treatment of mouse aorta with Ang II attenuated EDR, which was reversed by ER stress inhibitors. This finding agrees with recent studies showing inhibition of ER stress improves microvascular endothelial function in Ang II-induced hypertensive rats [2,8].

We further demonstrate that Ang II elevated the content of ER stress proteins, which was normalized by ER stress inhibitors. Phosphorylation of eIF2α is induced by activation of PERK. Furthermore, phosphorylation of eIF2α leads to selective translation of activating factor 4 (ATF4) which induces expressions of genes involved in ER-induced apoptosis (CHOP) and other genes including ER stress chaperone like Grp78. In this study, we mainly demonstrated the downstream signaling proteins following the action of two of the signaling pathways of ER stress. The addition of gene expression of CHOP, ATF4 and Grp78 shows the selective transcription of genes following phosphorylation of eIF2α. Although we have not demonstrated in this study, upregulation of CHOP and Grp78, either at gene level or protein level or both has been reported in Ang II-induced hypertensive model and inhibition of these markers improves Ang II-induced endothelial dysfunction [2, 8].

Blockade of AT1R reduces oxidative stress and improves endothelial function in diabetic mice [21] and in hypertensive rats and humans [22], suggesting a key role of increased oxidative stress in vascular dysfunction. We also demonstrate that AT1R blocker and antioxidant inhibit ER stress in human endothelial cells. In agreement with our present observation, antagonism of AT1R and scavenging of ROS decreased ER stress in non-vascular tissues [23].

Ang 1–7 opposes the biological effects of Ang II and confers protective action against Ang II-mediated pathologies [4,15]. The majority of the vaso-protective action of Ang 1–7 is attributed to its vasodilator effect, and its ability to improve NO bioavailability or to limit ROS formation [4,16]. However, whether this heptapeptide protects against Ang II-induced ER stress in blood vessels remains poorly understood. The present result demonstrated that Ang 1–7 reverses the increased vascular ER stress, decreased NO bioavailability, and impaired EDR upon Ang II stimulation.

The effects of Ang 1–7 are mediated by its interaction with the G-protein coupled Mas receptor [17, 24, 25, 26]. Ang 1–7 is likely to activate the Mas receptor as the Mas receptor antagonist A779 abolished the vascular protective effect of Ang 1–7 against ER stress. Activation of Mas receptor was shown to act as a physiological antagonist of AT1R [24]. Although we cannot exclude this possibility, we have further demonstrated that exogenous Ang 1–7 also inhibits the effect of ER stress inducer tunicamycin on NO production in HUVECs. In 2013, Uhal *et al*. reported that Ang 1–7 inhibited ER stress-induced apoptosis in alveolar epithelial cells, suggesting an inverse link between Ang 1–7 and ER stress. Furthermore, the effect of Ang 1–7 was abolished by the Mas receptor antagonist indicating the activation of Mas receptor mediates the beneficial effects of Ang 1–7 [27].

During ER stress, ROS production is increased via NADPH oxidase [4, 28] and Ang 1–7 inhibits the Ang II activation of NADPH oxidase [29]. Although not demonstrated, inhibition of ER stress by Ang 1–7 may decrease oxidative stress which contributes to the improved NO bioavailability. The present study examined the beneficial effects of Ang 1–7 against the *in vitro* Ang II-stimulated ER stress; this may not fully mimic the *in vivo* condition. Therefore, further study on chronic treatment of Ang 1–7 might help provide a better understanding of the role of Ang 1–7 against ER stress in Ang II-associated vascular dysfunction. Nevertheless, the present findings highlight the importance of Ang 1–7 to ameliorate ER stress by acting as the physiological antagonist to Ang II. Stimulating the Ang 1-7/ Mas receptor axis could be a potential therapeutic strategy to treat Ang II-related cardiovascular diseases related to ER stress.

## Supporting Information

S1 File Effect of ER stress inhibitors and AT1 receptor antagonist on endothelium dependent and independent relaxation. (DOC)
