# Correction: Angiotensin 1-7 Protects against Angiotensin II-Induced Endoplasmic Reticulum Stress and Endothelial Dysfunction via Mas Receptor

**DOI:** 10.1371/journal.pone.0147892

**Published:** 2016-01-22

**Authors:** Dharmani Murugan, Yeh Siang Lau, Chi Wai Lau, Mohd Rais Mustafa, Yu Huang

The third author’s name is spelled incorrectly. The correct name is: Chi Wai Lau. The correct citation is: Murugan D, Lau YS, Lau CW, Mustafa MR, Huang Y (2015) Angiotensin 1–7 Protects against Angiotensin II-Induced Endoplasmic Reticulum Stress and Endothelial Dysfunction via Mas Receptor. PLoS ONE 10(12): e0145413. doi:10.1371/journal.pone.0145413

There are errors in the affiliations for the third and fifth authors. Chi Wai Lau and Yu Huang are affiliated not only with 2, but also with Institute of Vascular Medicine, Shenzhen Research Institute, Chinese University of Hong Kong, Hong Kong, China.

## References

[1] Murugan D, Lau YS, Lau WC, Mustafa MR, Huang Y (2015) Angiotensin 1–7 Protects against Angiotensin II-Induced Endoplasmic Reticulum Stress and Endothelial Dysfunction via Mas Receptor. PLoS ONE 10(12): e0145413 doi: 10.1371/journal.pone.0145413 26709511 10.1371/journal.pone.0145413 PMC4692500

