# Supplementary material for: Angiotensin 1-7 Protects against Angiotensin II-Induced Endoplasmic Reticulum Stress and Endothelial Dysfunction via Mas Receptor
Source: PLoS One. 2015 Dec 28;10(12):e0145413. doi: 10.1371/journal.pone.0145413 (PMC4692500; doi:10.1371/journal.pone.0145413)
Supplement: S1 File — (DOC) [file pone.0145413.s001.doc]

S1 File

Figure A. SNP-induced endothelium-dependent relaxations (EDR) was not affected by the treatment with Ang II with or without PBA (10 µM), TUDCA (20 µM), losartan (3 µM, angiotensin type 1 receptor (AT1R) antagonist) and tempol (100 µM, superoxide dismutase mimetic) in mouse aortas. Data are mean ± S.E.M (n=4-6).

*

#

#

Figure B. Ang II-induced impairment of ACh-induced endothelium-dependent relaxations (EDR) was reversed by co-treatment with angiotensin type 1 receptor (AT1R) antagonist, losartan (3 µM); superoxide dismutase mimetic, tempol (100 µM) in mouse aortas. Data are mean ± S.E.M (n=5-6).^*^p<0.05 vs control; #p<0.05 vs Ang II.


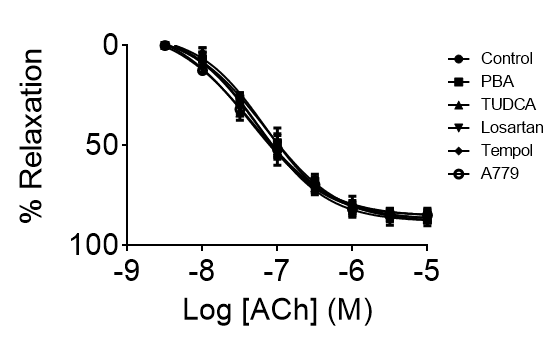


Figure C. ACh-induced endothelium-dependent relaxations (EDR) was not affected by treatment with PBA (10 µM), TUDCA (20 µM), losartan (3 µM, angiotensin type 1 receptor (AT1R) antagonist), tempol (100 µM, superoxide dismutase mimetic), A779 (10 µM, Mas receptor antagonist) in mouse aortas. Data are mean ± S.E.M (n=4-6).
